# Populations Can Be Essential in Tracking Dynamic Optima

**DOI:** 10.1007/s00453-016-0187-y

**Published:** 2016-08-26

**Authors:** Duc-Cuong Dang, Thomas Jansen, Per Kristian Lehre

**Affiliations:** 1 ASAP Research Group, School of Computer Science, University of Nottingham, Jubilee Campus, Wollaton Road, Nottingham, NG8 1BB UK; 2 Department of Computer Science, Aberystwyth University, Penglais Campus, Llandinam Building, Aberystwyth, SY23 3DB UK

**Keywords:** Runtime analysis, Population-based algorithm, Dynamic optimisation

## Abstract

Real-world optimisation problems are often dynamic. Previously good solutions must be updated or replaced due to changes in objectives and constraints. It is often claimed that evolutionary algorithms are particularly suitable for dynamic optimisation because a large population can contain different solutions that may be useful in the future. However, rigorous theoretical demonstrations for how populations in dynamic optimisation can be essential are sparse and restricted to special cases. This paper provides theoretical explanations of how populations can be essential in evolutionary dynamic optimisation in a general and natural setting. We describe a natural class of dynamic optimisation problems where a sufficiently large population is necessary to keep track of moving optima reliably. We establish a relationship between the population-size and the probability that the algorithm loses track of the optimum.

## Introduction

In a classical optimisation setting, so-called *static optimisation*, the focus is usually directed to finding an optimal or a high quality solution as fast as possible. In real-world optimisation, problem specific data may change over time, thus previously good solutions can lose their quality and must be updated or replaced. Automatic optimal control is a typical illustration of these situations, e. g. parameters of a machine can be set optimally under ideal conditions of a factory but they need to be adapted to changes in the real environment upon deployment. *Dynamic optimisation* is an area of research that is concerned with such optimisation problems that change over time. A specific characteristic is that it does not only focus on locating an optimal solution but also on tracking a moving optimum (see [7] for a definition).

It is often suggested that Evolutionary Algorithms (EAs), especially the ones with populations, are suitable for dynamic optimisation because a large population can contain different solutions which could be useful in the future [22]. However, theoretical demonstrations for how populations in dynamic optimisation can be essential are sparse and restricted to special cases. The ability of a very simple EA without a population, the $(1+1)$ EA, to track a target bitstring in a OneMax-like function is analysed in [4, 20]. The analysis has recently been extended from bitstrings to larger alphabets [11]. The influence of magnitude and frequency of changes on the efficiency of the $(1+1)$ EA in optimising a specifically designed function was investigated in [19], showing that some dynamic optimisation problems become easier with higher frequency of change. The analysis of the $(1+\lambda )$ EA that uses a larger offspring population but still not a real population on a simple lattice problem is presented in [9]. The efficiencies of specific diversity mechanisms when using an actual population were compared in [18]. This was done for a specific example function (introduced by [19]) and considering low frequency of changes. It was shown in [12] that a Min-Max Ant System (MMAS) can beat the $(1+1)$ EA in a deterministic dynamic environment. The comparison was later extended to general alphabets and to the $(\mu +1)$ EA that preserves genotype diversity [15]. With that particular setting of the $(\mu +1)$ EA, the size of the alphabets defines a threshold on the parent population size $\mu$ so that the algorithm is able to track and reach the optimal solution in polynomial time. The result was also extended to the single-destination Shortest Path Problem [14]. Comparisons were also made between EAs and Artificial Immune System (AIS) on a OneMax-like problem with the dynamic being periodic [10].

Considering the existing analyses we can in summary note two shortcomings that leave the impression that important fundamental questions about dynamic optimisation are still not answered satisfactorily. One shortcoming is the concentration on simple evolutionary algorithms and other search heuristics that do not make use of an actual population. Clearly, the advantages of a population-based approach cannot be explored and explained this way. The other is that many studies consider very complex dynamic environments that make it hard to see the principal and fundamental issues. Therefore, the fundamental question why even a simple population without complicated diversity mechanisms can be helpful in dynamic environments requires more attention.

Motivated by the above facts, we will use a simple argument considering a very general class of dynamic functions to show that a population is essential to keep track of the optimal region. We define our function class on the most often used search space, bit strings of a fixed length. However, it is not difficult to extend the function class to be defined for any finite search space $\mathcal {X}$ and any *unary* mutation operator $p_\mathrm {mut} :\mathcal {X} \rightarrow \mathcal {X}$. The class is called $(cn,\rho )$ *-stable* on $\mathcal {X}$ with respect to $p_\mathrm {mut}$, where *n* is the required number of bits to specify a search point of $\mathcal {X}$ and *c* and $\rho$ are positive constants independent of *n*. The function class is only restricted by the probability of recovering the optimal region via the mutation operator $p_\mathrm {mut}$ (see Definition 4). The definition of the function class does not refer explicitly to other function characteristics, such as the topology or the fitness values of the set of optimal solutions, or the distribution of fitness values of the set of non-optimal solutions.

We will use the *Moving Hamming Ball* function from [2] as an illustrative example over the search space $\{0,1\}^{n}$ and with respect to the bitwise mutation operator. We also use this specific function to argue that an approach based on a single individual, such as the $(1+1)$ EA, is inefficient in tracking the optimal region in spite of being equipped with the same mutation operator. On the other hand, we show that a population-based algorithm with a sufficiently large population can efficiently track the moving optimal region of any dynamic function of the class defined for any given finite search space.

The remainder of the paper is organised as follows. The next section first gives a formal description of dynamic optimisation and efficient tracking, then the class of dynamic functions that we consider is described with an example function. Next, we consider the $(1+1)$ EA and RLS on the function class and provide an analysis to serve as an example how search heuristics based on single solutions are not able to track the optimal solutions over time. The efficiency of population-based algorithms is then explained by proving a positive result about their performance. Here, we use the setting of non-elitist populations and show that, with a sufficient selective pressure, the ability of the population to track the moving optimal region is overwhelmingly high with respect to the population size. On the other hand, as a consequence of a fair comparison to a single-individual approach, the population must not be too big in order to capture the frequency of changes. Finally, we summarise, conclude and point out directions for future research.

The paper uses the following notation and terminology. For any positive integer *n*, define $[n]{:=}\{1,2,\ldots ,n\}$. The natural logarithm is denoted by $\ln (\cdot )$, and the logarithm to the base 2 is denoted by $\log (\cdot )$. The Hamming distance is denoted by $\mathrm {H}(\cdot ,\cdot )$ and the Iverson bracket is denoted by $[\cdot ]$. We use $\mathbbm {1}_{A}$ to denote the indicator function of a set *A*, i.e. $\mathbbm {1}_{A}(x) = 1$ if $x \in A$, and 0 otherwise. For a given bitstring $x \in \{0,1\}^n$, the Hamming ball around *x* with radius *r* is denoted by $\mathrm {B}_r(x):=\{y \in \{0,1\}^n \mid \mathrm {H}(x,y) \le r\}$. The bitstring containing *n* one-bits and no zero-bits is denoted $1^n$. An event is said to occur with overwhelmingly high probability (w. o. p.) with respect to a parameter *n*, if the probability of the event is bounded from below by $1-e^{-\varOmega (n)}$.

## A General Class of Dynamic Functions

Before defining the class of $(\kappa ,\rho )$-stable dynamic functions which will be studied in this paper, we first formalise our notion of dynamic optimisation, and we define what we mean when saying that a dynamic search heuristic tracks a moving optimal region efficiently.

### A Formal Description of Dynamic Optimisation

We focus on optimisation of pseudo-Boolean functions with discrete-time dynamics, as formalised below. Note that our formalisation can be generalised to any finite search space $\mathcal {X}$, e.g. replacing $\{0,1\}^n$ with $\mathcal {X}$, and our results for population-based algorithms also hold for this generalisation.

#### Definition 1

A dynamic function *F* is a random sequence of functions $(f_t)_{t\in \mathbb {N}},$ where $f_t:\{0,1\}^n\rightarrow \mathbb {R}$ for all $t\in \mathbb {N}$. The optimal regions associated with *F* is the sequence $(\mathrm {OPT}_{t})_{t\in \mathbb {N}}$, where $\mathrm {OPT}_{t}=\arg \max _x f_t(x)$.

The perhaps simplest, non-trivial example of a dynamic function is a periodic function which deterministically alternates between two functions, say $g_1$ and $g_2$, such that $f_{2i-1}=g_1$ and $f_{2i}=g_2$ for all $i\in \mathbb {N}$. We will consider more complex dynamic functions, where the sequence of functions is random and non-periodic. Although the sequence of functions in a dynamic function is random, each individual function is deterministic, i.e., we do not consider dynamic optimisation with noisy functions.

In this paper, we do not make any assumption about the changes of the function and the speed of the algorithm. It has been pointed out that it is important to consider the relationship between the speed of the execution platform where the algorithm runs and the speed of change of the function because this has significant influence on the performance [10]. Almost all studies assume that the function cannot change within one generation of the algorithm. The only exception we are aware of is a paper by Branke and Wang [1] who analyse a (1, 2) EA. We follow this idea but consider a much broader class of algorithms.

When applying a search heuristic to a dynamic function, we therefore have to consider two time lines: the first is associated with the evolution of the dynamic function, and the second is associated with the search points generated by the heuristic. Following the convention from black-box complexity [6], we assume that the function evaluations are the most expensive operations, for sake of the analysis becoming the basic time steps of an algorithm. The time consumed by all other operations, such as sampling an individual or applying a mutation operator, is assumed to be negligible. We connect the two time-lines by assuming that every time the heuristic queries a search point, the time-line of the dynamic function increases by one. We allow dynamic search heuristics some flexibility in that search points can be queried not only with respect to the most recent function $f_t$, but also with respect to past functions. For example, the individuals in a population can be evaluated with respect to one particular time. We also assume that the decisions made by the search heuristic does not influence the dynamic of the function. The dynamic optimisation-scenario we have described is summarised in the following definition.

#### Definition 2

A dynamic search heuristic is an algorithm which given a search history $\left( (x_j,i_j,f_{i_j}(x_j)\right) _{j\in [t-1]}$ of $t-1$ elements in $\{0,1\}^n\times \mathbb {N}\times \mathbb {R}$, selects a search point $x_t\in \{0,1\}^n$ and an evaluation time $i_t\in [t]$, and evaluates $f_{i_t}(x_t)$.

An element $(x_t, i_t, f_{i_t}(x_t))$ in a search history describes the search point $x_t$ queried by the algorithm in step *t*, the time point $i_t\le t$ with which the search point is evaluated, and the corresponding function value $f_{i_t}(x_t)$. We can now formalise the notion of *efficient tracking* of optima.

#### Definition 3

A search heuristic is said to *efficiently track the optima* of a dynamic function *F* if there exist $t_0,\ell \in {{\mathrm{poly}}}(n)$ and constants $c,c^{\prime }>0$ such that

$$\begin{aligned} \min _{t_0<t<e^{cn}}\Pr \left( \sum _{i=t}^{t+\ell }\mathbbm {1}_{\{x_i\in \mathrm {OPT}_{i}\}} \ge c^{\prime }\ell \right) \ge 1-e^{-\varOmega (n)}, \end{aligned}$$

where $(x_t)_{t\ge 0}$ is the sequence of search points queried by the heuristic, and $(\mathrm {OPT}_{t})_{t\ge 0}$ is the sequence of optimal search points of function *F*.

Informally, Definition 3 means that the algorithm queries optimal search points frequently. More precisely, within every sub-interval of length $\ell$ within the exponentially long time interval from $t_0$ to $e^{cn}$, a constant fraction of the queried search points are optimal. Note that the optimality of a search point is defined with respect to the query time, and regardless of the function with which the algorithm evaluates the search point. The constraint $\ell \in {{\mathrm{poly}}}(n)$ on the length of sub-intervals guarantees that the time between generation of two optimal search points is bounded from above by a polynomial. It is clear from the definition that an algorithm is inefficient if with a sufficiently high probability, e.g. at least constant, it loses track of the optimal region and does not recover it within a polynomial number of steps.

### A Class of Stable Dynamic Functions

The class of $(\kappa ,\rho )$-stable dynamic functions with respect to a variation operator is defined as follows.

#### Definition 4

Let $\phi :\{0,1\}^n \rightarrow \{0,1\}^n$ be any *unary* variation operator, and $\kappa \in \mathbb {N}$, $\rho \in (0,1)$. If there exist constants $c,c^{\prime }>0$ such that with probability at least $1 - e^{-c^{\prime }\kappa }$, the optimal regions $(\mathrm {OPT}_{t})_{t\in \mathbb {N}}$ of a function *F* satisfy for all time points *t* and $t^{\prime }$ with $0< t< t^{\prime } \le t+\kappa <e^{c\kappa }$, and for all search points $x\in \mathrm {OPT}_{t}$,

$$\begin{aligned} \Pr \left( \phi (x) \in \mathrm {OPT}_{t^{\prime }}\right) \ge \rho \end{aligned}$$

then *F* is called $(\kappa ,\rho )$-stable with respect to $\phi$.

**Fig. 1:**
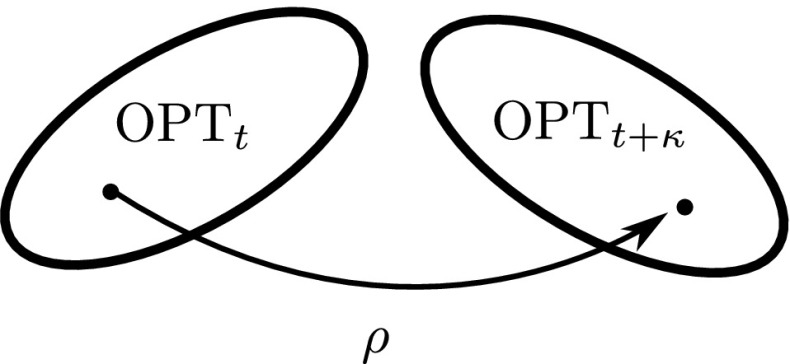
Illustration of a $(\kappa ,\rho )$-stable dynamic function, in which any search point in the optimal region of time *t* can be mutated into the optimal region of time $t+\kappa$ with probability at least $\rho$

Definition 4 covers a large class of dynamic optimisation functions for any given pair of parameters $(\kappa ,\rho )$. The optimal regions over time can take many shapes, including disconnected pieces over $\{0,1\}^n$ as long as the distances between them and the cardinality of the intersections allow the probabilistic condition to hold. Figure 1 illustrates the required condition.

Given an operator $\phi$, we focus on the class of $(cn,\rho )$-stable functions where *c* and $\rho$ are positive constants. We will show that a population-based algorithm with a sufficiently large population and a sufficiently strong selection pressure can track the optimal region of any function in the class efficiently. The next section gives an example function of the class for $\phi$ being bitwise mutation and explains how it fits within the framework of $(cn,\rho )$-stable function. We will then use the example function to argue that algorithms that base their search on a single individual, such as the $(1+1)$ EA, can be inefficient.

### Example of a Stable Pseudo-Boolean Function

We consider the Moving Hamming Ball function as described in [2]. The static version of the function has the following form.

#### Definition 5

The Hamming Ball function around a target bitstring $x^{*}$ and a radius *r* is defined as,

$$\begin{aligned} \mathrm {HB}^{r,x^{*}}(x)&={\left\{ \begin{array}{ll} 1&{}\text {if }\mathrm {H}(x, x^{*}) \le r, \\ 0&{}\text {otherwise}. \end{array}\right. } \end{aligned}$$

It suffices to change $x^{*}$ in sequential steps to create a dynamic version from the static one. We use the following dynamic setting for the function: the points in time when the target $x^{*}$ is changed are determined by a sequence of random variables drawn from a Poisson distribution.

#### Definition 6

Let $(X_i)_{i\in \mathbb {N}}$ be a sequence of random variables independently sampled from a Poisson distribution with parameter $\theta$, $X_i \sim {{\mathrm{Pois}}}(\theta )$, $\ell$ be some integer in [*n*], and $\left( x^{*}_i\right) _{i\in \mathbb {N}}$ be a sequence of bitstrings generated by

$$\begin{aligned} x^{*}_i&= {\left\{ \begin{array}{ll} 1^n &{} \text { if } i = 0, \\ \sim {{\mathrm{Unif}}}\left( \left\{ y \left| \mathrm {H}\left( x^{*}_{i-1},y\right) = \ell \right\} \right) \right. &{} \text { otherwise. } \end{array}\right. } \end{aligned}$$

The Moving Hamming Ball ($\mathrm {MHB}$) function with parameters *r*, $\ell$, and $\theta$ is defined as

$$\begin{aligned}&\mathrm {MHB}^{r,\ell ,\theta }_t(x)= \mathrm {HB}^{r,x^{*}(t)}(x) \\&\text {where } x^{*}(t)= x^{*}_{\max \{j \mid \sum _{i=1}^{j} X_i \le t\}}. \end{aligned}$$

The $\mathrm {MHB}$ function fits within the stability framework of Definition 4 with respect to the bitwise mutation operator $p_\mathrm {mut}^\mathrm {EA}$. This variation operator, which has a parameter $\chi \in [0,n]$, flips each position in the bitstring independently with probability $\chi /n$. Hence, the probability of mutating a bitstring $x\in \{0,1\}^n$ into a bitstring $y\in \{0,1\}^n$ is

$$\begin{aligned} \Pr \left( y=p_\mathrm {mut}^\mathrm {EA} (x)\right) = \left( \frac{\chi }{n}\right) ^{H(x,y)}\left( 1-\frac{\chi }{n}\right) ^{n-H(x,y)}. \end{aligned}$$

#### Lemma 7

For all positive constants *d*, $\chi$ and $\varepsilon$, the function $\mathrm {MHB}^{r,\ell ,\theta }$ is $\left( \frac{\theta }{1+d}, \left( \frac{r\chi }{n\ell }\right) ^{\ell } e^{-(1+\varepsilon )\chi }\right)$-stable with respect to the bitwise mutation operator $p_\mathrm {mut}^\mathrm {EA}$ with parameter $\chi$.

#### Proof

For any given time *t*, let *X* be the random variable associated with the number of time steps in the future that the target bitstring will be changed. If we pick $\kappa := \theta /(1+d)$, then it is clear that within the next $\kappa$ time steps, there will be more than one change of the target bitstring if and only if $X \le \kappa$. It follows from Lemmas 18 and 19 that

$$\begin{aligned} \Pr \left( X \le \kappa \right)&\le e^{-\theta }\left( \frac{e\theta }{\kappa }\right) ^{\kappa } = e^{-(1+d)\kappa }\left( (1+d)e\right) ^{\kappa } \\&= e^{-d\kappa }(1+d)^{\kappa } \le \exp \left( -d\kappa + \kappa \cdot \frac{d}{2}\cdot \frac{d+2}{d+1}\right) \\&= \exp \left( -\frac{\kappa }{2}\left( \frac{d^2}{d+1}\right) \right) . \end{aligned}$$

It suffices to pick the constant $\upsilon {:=} \frac{d^2}{2(d+1)}$ so that with a probability of at least $1 - e^{-\upsilon \kappa }$, there is at most one change to the target function within the next $\kappa$ time steps. Under that condition, it holds for all $t^{\prime }\in [t, t+\kappa ]$ and for all $x \in \mathrm {B}_r(x(t)) =: \mathrm {OPT}_{t}$, that $\mathrm {H}(x, x(t^{\prime })) = r + \ell ^{\prime }$ for some $\ell ^{\prime }\in \{0\} \cup [\ell ]$.

In the case that $\ell ^{\prime }=0$, e.g. the target does not move or it moves closer to *x*, it suffices to not flip any of the $n - r$ bits. For any constant $\varepsilon$, it holds for all $n \ge (1+1/\varepsilon )\chi$ that

$$\begin{aligned} \Pr \left( p_\mathrm {mut}^\mathrm {EA} (x) \in \mathrm {B}_r(x(t^{\prime }))\mid \ell ^{\prime }=0\right)&\ge \left( 1 - \frac{\chi }{n}\right) ^{n-r} \ge \left( 1 - \frac{\chi }{n}\right) ^{\left( \frac{n}{\chi }-1\right) \chi \left( 1 + \frac{\chi }{n - \chi }\right) } \\&\ge e^{-(1+\varepsilon )\chi }. \end{aligned}$$

In the case that $\ell ^{\prime }>0$, it suffices to recover the $\ell ^{\prime }$ bits among the $r + \ell ^{\prime }$ mismatched ones, so

$$\begin{aligned} \Pr \left( p_\mathrm {mut}^\mathrm {EA} (x) \in \mathrm {B}_r(x(t^{\prime }))\mid \ell ^{\prime }>0\right)&\ge \left( {\begin{array}{c}r + \ell ^{\prime }\\ \ell ^{\prime }\end{array}}\right) \left( \frac{\chi }{n}\right) ^{\ell ^{\prime }} \left( 1 - \frac{\chi }{n}\right) ^{n - \ell ^{\prime }} \\&\ge \left( \frac{r + \ell ^{\prime }}{\ell ^{\prime }}\right) ^{\ell ^{\prime }}\left( \frac{\chi }{n}\right) ^{\ell ^{\prime }} e^{-(1+\varepsilon )\chi } \\&> \left( \frac{r\chi }{\ell ^{\prime }n}\right) ^{\ell ^{\prime }}e^{-(1+\varepsilon )\chi } \ge \left( \frac{r\chi }{\ell n}\right) ^{\ell }e^{-(1+\varepsilon )\chi }. \end{aligned}$$

Note that $\mathrm {OPT}_{t^{\prime }} := \mathrm {B}_r(x(t^{\prime }))$, hence

$$\begin{aligned} \Pr \left( p_\mathrm {mut}^\mathrm {EA} (x) \in \mathrm {OPT}_{t^{\prime }}\right) \ge \left( \frac{r\chi }{\ell n}\right) ^{\ell }e^{-(1+\varepsilon )\chi } =: \rho \end{aligned}$$

and $\mathrm {MHB}^{r,\ell ,\theta }$ is $(\kappa ,\rho )$-stable with respect to $p_\mathrm {mut}^\mathrm {EA}$. $\square$

It is not difficult to see that the stability condition of the function class still holds with the following relaxations:

- The fitness of the solutions inside the Hamming ball changes when the target string moves,
- The fitness of the solutions outside the current Hamming Ball can be distributed differently, as long as they are less than the current optimal fitness,
- The moving step $\ell$ is relaxed to be sampled from any discrete distribution over $[\ell ]$ in each change of the target bitstring.

We will not consider these relaxations as they are not required to distinguish between the effectiveness of single-individual and population-based evolutionary algorithms.

## Algorithms

We will compare the performance of population-based and single-individual based evolutionary algorithms. In this section we first define these classes of algorithms.

We are considering dynamic optimisation problems and, as mentioned in the introduction and discussed by Jansen and Zarges [10], it is important to clarify how the algorithms deal with change of the fitness functions, in particular if this happens during one generation. In this paper, we consider algorithms that make use of consistent comparisons when applying on a dynamic function: when an algorithm has to make fitness comparisons on a set of solutions, it will first make a *static copy* of the dynamic function and evaluate the solutions on this copy. This approach corresponds to an implementation where the necessary data to evaluate the optimisation function is collected before evaluating a set of solutions. Meanwhile the real function may have changed more or less depending on the number of solutions in the set.

We first consider the single-individual approach described in Algorithm 1. The algorithm keeps a current search point *x*. In each iteration, it produces a new candidate solution $x^{\prime }$, and compares it with the current search point using the same function. Hence, static copies of the dynamic function are made in every two time steps. This corresponds to a frequent update of the dynamic function. We let $p_\mathrm {mut}$ be the bitwise mutation operator $p_\mathrm {mut}^\mathrm {EA}$ described in Sect. 2.3, and obtain the well-known $(1+1)$ EA [5]. However, the result can be easily generalised to other mutation operators, such as the single-bit flip operator used in the RLS algorithm.
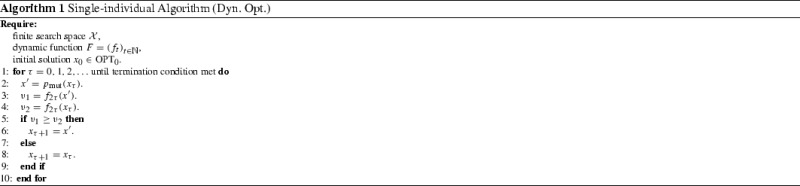


We are mostly interested in the influence of the population size, designated by the parameter $\lambda$, on the ability of a population-based algorithm to track the moving optimal region. We focus on the non-elitist setting as described in Algorithm 2. The algorithm uses a unary variation operator denoted by $p_\mathrm {mut}$, no crossover operator, and an unspecified selection mechanism $p_\mathrm {sel}$. The selection mechanism is any random operator $p_\mathrm {sel}$ that given a population *P* and access to a fitness function returns one of the individuals in *P*. By specifying different $p_\mathrm {sel}$ and $p_\mathrm {mut}$, Algorithm 2 can instantiate a large number of population-based search heuristics, such as the ($\mu ,\lambda$) EA. The number of search points $\lambda$ produced in each round is the only parameter that appears in the description of Algorithm 2. The ($\mu ,\lambda$) EA fits within this framework by making sure that the selection in line 4 only takes into account the $\mu$ best of the $\lambda$ search points created in the last round.

The algorithm maintains a population $P_\tau$ of $\lambda$ individuals which during one generation (steps 2–6) is replaced by a newly created population $P_{\tau +1}$ of the same size. As for the $(1+1)$ EA, we assume that the initial population $P_0$ is contained in the first optimal region $\mathrm {OPT}_{0}$. Each individual in the next population $P_{\tau +1}$ is created by first making a copy *x* of one parent individual which is selected from the current population (step 4 selection), then modifying the copy using $p_\mathrm {mut}$ operator (step 5, mutation).

When selecting individuals, the algorithm must take into account that multiple changes to the fitness function can occur during one generation. Here, we assume that the algorithm makes a static copy of the fitness function $f_{\tau \lambda }$ at the beginning of each generation, i.e. at time $\tau \lambda$. The selection mechanism $p_\mathrm {sel}$ compares all individuals in a generation using the static copy. Note that if the population size $\lambda$ is too large with respect to the problem parameter $\theta$ (which controls the frequency of change of the dynamic function), then the optimal region may change several times between two consecutive generations. Hence, the population size should not be too large. However, we will show in the next section that a sufficiently large population is also essential to keep the population within the optimal region.

The result for populations will be first shown for any finite search space and any mutation operator $p_\mathrm {mut}$ because the class of dynamic function is defined with respect to the operator $p_\mathrm {mut}$. Then we will use $p_\mathrm {mut}^\mathrm {EA}$ over $\{0,1\}^n$ as a specific example.
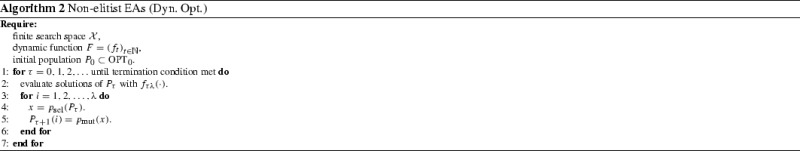


Although Algorithm 2 can use any selection mechanism $p_\mathrm {sel}$, we are looking for choices of $p_\mathrm {sel}$ that allows the algorithm to track optima efficiently. Formally, $p_\mathrm {sel}$ applied on finite populations of size $\lambda$ is represented by the transition matrix $p_\mathrm {sel} :[\lambda ] \times \mathcal {X}^\lambda \rightarrow [0,1]$, where $p_\mathrm {sel} (i\mid P)$ represents the probability of selecting individual *P*(*i*), i.e. the *i*-th individual, of *P*. We also write $x=p_\mathrm {sel} (P)$, e.g. in the algorithm, to express that *x* is sampled from the distribution over *P* given by $p_\mathrm {sel} (\cdot ,P)$. We use $x_{(i)}$ to denote the *i*th best individual of *P*, or the so-called $(i/\lambda )$-ranked individual. Similar to [3, 13], we characterise $p_\mathrm {sel}$ by the cumulative selection probability.

### Definition 8

([13]). Given a fitness function $f:\mathcal {X} \rightarrow \mathbb {R}$, the *cumulative selection probability* $\beta$ associated with selection mechanism $p_\mathrm {sel}$ is defined on *f* for all $\gamma \in (0,1]$ and a $P\in \mathcal {X}^\lambda$ by

$$\begin{aligned} \beta (\gamma ,P) := \sum _{i\in [\lambda ]} p_\mathrm {sel} (i \mid P) \cdot \left[ f(P(i)) \ge f(x_{(\lceil \gamma \lambda \rceil )}) \right] . \end{aligned}$$

Informally, $\beta (\gamma ,P)$ is the probability of selecting an individual with fitness at least as good as that of the $\gamma$-ranked individual, assuming that *P* is sorted according to fitness values. We are interested in a lower bound function of $\beta (\gamma ,P)$. Most often this lower bound is independent of *P*, in which case we simply write it as a function of $\gamma$ only, i. e. as $\beta (\gamma )$.

## Performance Analysis

In this section, we first show that single-individual approaches are inefficient in tracking moving optima on at least one example function of the class, precisely on $\mathrm {MHB}^{r,\ell ,\theta }$. Then we prove a general result that an appropriately parameterised population-based algorithms can efficiently track the moving optima of any function in the class.

### Inefficiency of a Single Individual

In this section, we will show that the $(1+1)$ EA spends an exponential fraction of its time outside the optimal region of a $\mathrm {MHB}^{bn,\ell ,cn}$ function, for a sufficiently small constant *b*, any constant $c>0$ and any $\ell \ge 1$. That is to say the algorithm is inefficient even in tracking such a stable function.

To prove such a result, we have to analyse the behaviour of the algorithm both inside and outside the moving Hamming ball: We assume that the algorithm starts at the center of the first optimal region and show that after some initial time, whenever the center of the ball moves, there is a constant probability that the $(1+1)$ EA will memorise a search point outside of the new ball; Whenever the algorithm is outside of the optimal region there is also a constant probability that the memorised search point will drift away from the optimal region (eventually get lost), before an optimal solution inside the new ball is discovered. Since the changes to the function happens within an expected polynomial number steps, we can conclude that with a high probability, the $(1+1)$ EA only spends a polynomial number of time steps inside the moving Hamming ball.

We start with the first argument, the behaviour of the algorithm inside the Hamming ball. We notice that the changes induced by the dynamics of the fitness function strongly drag the target away from the current memorised search point, however this does not happen in every iteration. In every iteration, the changes by mutation drive the memorised solution away from the center of the current Hamming ball, but the elitist selection also keeps the memorised solution from falling outside. We have the following analysis of the drift.

We consider the process $(X_t)_{t\in \mathbb {N}}$, where $X_t$ is the Hamming distance to the border of the optimal region of $\mathrm {MHB}^{bn,\ell ,cn}$ at time *t*, i.e. $X_t = r - \mathrm {H}(x^{*}(t), x_\tau )$. The process starts with $X_t = r$, e.g. exactly at the center of the Hamming ball. Given $X_t = i$, define $\varDelta (i){:=}X_{t} - X_{t+1}$, then $\mathbf {E}\left[ \varDelta (i)\mid X_{t}=i\right]$ is the drift towards the border at time *t* and where $X_t = i$.

First of all, the dynamic now only kicks in every *cn* time steps in expectation. Also, the contributing drift is positive. For example, if the dynamic kicks in, let *Z* be the number of bits being corrected by the dynamic, then we have $Z \sim {{\mathrm{Hypergeo}}}(n,n-i,\ell )$, and the contributing drift is $\mathbf {E}\left[ \ell - 2Z\right] = \ell (1 - 2i/n) > 0$ for any $r/n<1/2$.

We now compute the drift by mutation at time *t* and where $X_t = i > 0$. Let *X* and *Y* be the number of bits being corrected and being messed up respectively by the mutation, so $X \sim {{\mathrm{Bin}}}(r - i,\chi /n)$, $Y \sim {{\mathrm{Bin}}}(n-(r - i),\chi /n)$ and the two variables are independent. Note that for all integers $X \ge 0$, $Y \ge 0$ and $i \ge 1$, it holds

$$\begin{aligned} \varDelta (i)&= (Y - X) \cdot \mathbbm {1}_{\{Y - X \le i\}} = Y \cdot \mathbbm {1}_{\{Y \le i + X\}} - X \cdot \mathbbm {1}_{\{X \ge Y - i\}} \\&\ge Y \cdot \mathbbm {1}_{\{Y \le 1\}} - X = \mathbbm {1}_{\{Y = 1\}} - X =: \varDelta _1(i). \end{aligned}$$

Thus for $i > 0$, $\varDelta (i)$ stochastically dominates $\varDelta _1(i)$ and we also have

$$\begin{aligned} \mathbf {E}\left[ \varDelta _1(i) \mid X_t = i\right]&= \mathbf {E}\left[ \mathbbm {1}_{\{Y = 1\}} \right] - \mathbf {E}\left[ X\right] \\&= \left( {\begin{array}{c}n - (r - i)\\ 1\end{array}}\right) \left( \frac{\chi }{n}\right) \left( 1 - \frac{\chi }{n}\right) ^{n-(r - i) - 1} - \frac{i\chi }{n} \\&\ge \chi \left( \left( 1-\frac{r - i}{n}\right) e^{-(1+\varepsilon )\chi } - \frac{r - i}{n}\right) \\&> \chi \left( \left( 1-\frac{r}{n}\right) e^{-(1+\varepsilon )\chi } - \frac{r}{n}\right) \\&= \chi \left( \left( 1-b\right) e^{-(1+\varepsilon )\chi } - b\right) \end{aligned}$$

for any constant $\varepsilon >0$. Therefore, with any constant $b < 1/(1+ 2 e^\chi )$, we have that $b \le (1 - b)e^{-(1+\varepsilon )\chi }/2$. Hence, for $i > 0$ we have at least a constant drift away from the center

$$\begin{aligned} \mathbf {E}\left[ \varDelta _1(i) \mid X_t = i\right] > \left( \frac{\chi }{2\cdot e^{(1+\varepsilon )\chi }}\right) \left( 1 - b\right) =: \delta . \end{aligned}$$

The only position where we have a drift toward the center is the one at the border, e.g. $X_t = 0$. However, this is not a strong drift. When the target bitstring does not move in the next iteration, we have $- \varDelta (0) = (X - Y)\cdot \mathbbm {1}_{\{Y - X \le 0\}} \le X \cdot \mathbbm {1}_{\{X \ge Y\}} \le X$, then the negative drift is no more than

$$\begin{aligned} \mathbf {E}\left[ X \right] = \frac{r \chi }{n} =: \eta . \end{aligned}$$

In summary, we get the drift by mutation:

1 $$\begin{aligned} \mathbf {E}\left[ \varDelta (i) \cdot \mathbbm {1}_{\{X_t> 0\}} \mid X_t = i\right]&\ge \delta \cdot \mathbbm {1}_{\{X_t > 0\}}\end{aligned}$$

2 $$\begin{aligned} \mathbf {E}\left[ \varDelta (i) \cdot \mathbbm {1}_{\{X_t = 0\}} \mid X_t = i\right]&\ge -\eta \cdot \mathbbm {1}_{\{X_t = 0\}} \end{aligned}$$

It is then suggested that the equilibrium state of the memorised search point is around the border. Furthermore, we can quantify the expected fraction of time that the search point is found at the border, using the following tool.

#### Lemma 9

Given a stochastic process $(X_t)_{t\ge 0}$ over a state space $\mathbb {N}$, and two constants $\eta ,\delta \in \mathbb {R}_+$ such that

- $$\mathbf {E}\left[ X_{t+1} \cdot \mathbbm {1}_{\{X_t=0\}}\mid X_t\right] \le \eta \cdot \mathbbm {1}_{\{X_t=0\}}$$, and
- $$\mathbf {E}\left[ X_{t+1} \cdot \mathbbm {1}_{\{X_t> 0\}}\mid X_t\right] \le (X_t-\delta ) \cdot \mathbbm {1}_{\{X_t> 0\}}$$,

then for all $t\ge 1$

$$\begin{aligned} \sum _{i=0}^{t-1}\Pr \left( X_t=0\right) \ge \frac{\delta t-X_0}{\delta +\eta }. \end{aligned}$$

#### Proof

Define $p_i=\Pr \left( X_i=0\right)$. For all $t\ge 1$, it holds

$$\begin{aligned} \mathbf {E}\left[ X_t\right]&= \mathbf {E}\left[ \mathbbm {1}_{\{X_{t-1}=0\}} \cdot X_{t}\right] +\mathbf {E}\left[ \mathbbm {1}_{\{X_{t-1}>0\}} \cdot X_{t}\right] \\&=\mathbf {E}\left[ \mathbf {E}\left[ \mathbbm {1}_{\{X_{t-1}=0\}} \cdot X_{t}\mid X_{t-1}\right] \right] +\mathbf {E}\left[ \mathbf {E}\left[ \mathbbm {1}_{\{X_{t-1}>0\}} \cdot X_{t}\mid X_{t-1}\right] \right] \\&\le \mathbf {E}\left[ \eta \cdot \mathbbm {1}_{\{X_{t-1}=0\}} \right] + \mathbf {E}\left[ (X_{t-1}-\delta ) \cdot \mathbbm {1}_{\{X_{t-1}>0\}}\right] \\&=\eta p_{t-1}-\delta (1-p_{t-1})+\mathbf {E}\left[ X_{t-1} \cdot \mathbbm {1}_{\{X_{t-1}>0\}} \right] \\&=\eta p_{t-1}-\delta (1-p_{t-1})+\mathbf {E}\left[ X_{t-1}\right] . \end{aligned}$$

It follows that

$$\begin{aligned} \mathbf {E}\left[ X_t\mid X_0\right] \le X_0-t\delta +(\delta +\eta )\sum _{i=0}^{t-1}p_t. \end{aligned}$$

Finally, since $$\mathbf {E}\left[ X_t\mid X_0\right] \ge 0$$

$$\begin{aligned} \sum _{i=0}^{t-1}p_t \ge \frac{t\delta -X_0}{\delta +\eta }. \end{aligned}$$

$$\square $$

The following lemma considers non-negative, integer-valued stochastic processes with positive drift at most $\eta$ in state 0, and negative drift at least $\delta$ elsewhere. It provides a lower bound on the probability of such a process being in state 0 after some time.

#### Lemma 10

Let $(X_t)_{t\ge 0}$ be any stochastic process with support in $\{0\}\cup [r]$ for some fixed $r\in \mathbb {N}$, which satisfies the properties of Lemma 9 for some $\delta ,\eta \in \mathbb {R}_+$. Then for any random variable $T_1\ge \lceil 2r/\delta \rceil$ which is independent of $(X_t)_{t\ge 0}$, it holds

$$\begin{aligned} \Pr \left( X_{T_1}=0\right) \ge \frac{\delta }{2(\delta +\eta )}. \end{aligned}$$

#### Proof

Choose $t:= \lceil 2r/\delta \rceil$, and define $Y_i:=X_{T_0+i}$ where $T_0:=T_1-T$ and $T\sim {{\mathrm{Unif}}}( \{0\}\cup [t-1])$, i.e., we consider the process $X_t$ from a random starting point $T_0\ge 0$. Due to independence between $T_1, T$, and $(X_t)_{t\ge 0}$, we have

$$\begin{aligned} \Pr \left( X_{T_1}=0\right)&= \sum _{i=0}^{t-1}\Pr \left( Y_i=0 \wedge T_0+i=T_1\right) \\&= \sum _{i=0}^{t-1}\Pr \left( Y_i=0\right) \Pr \left( T = i\right) \\&= \sum _{i=0}^{t-1}\frac{1}{t}\Pr \left( Y_i=0\right) . \end{aligned}$$

Lemma 9 applied to $(Y_t)_{t\ge 0}$ now implies

$$\begin{aligned} \sum _{i=0}^{t-1}\frac{1}{t}\Pr \left( Y_i=0\right) \ge \frac{\delta -Y_0/t}{\delta +\eta } \ge \frac{\delta }{2(\delta +\eta )}. \end{aligned}$$

$$\square $$

We now show that once the $(1+1)$ EA has lost track of the optimal region it will take a long time to recover. We assume that the objective function is $\mathrm {MHB}^{bn,\ell ,cn}$ with radius $r = bn \ll n/2$, i.e. $b \le (1/2) - \kappa$ for some constant $\kappa > 0$ (note that *b* can depend on *n*). The first step in this proof is to show that with not too small probability the $(1+1)$ EA ends up far away (more specifically, in a linear distance) from the Hamming ball before recovering it.

#### Lemma 11

Given $o \in \{0,1\}^n$, let $(x_t)_{t \ge 0}$ be a sequence of random bit strings such that $x_t = p_\mathrm {mut}^\mathrm {EA} (x_{t-1})$ and $x_0 \in \mathrm {B}_{r+1}(o)$ for some $r = bn \ll n/2$, i.e. $0< b \le 1/2 -\kappa$ for some $\kappa >0$. For any $d \in \mathbb {N}_{+}$, define $T_{r, d} := \inf \left\{ t \mid \mathrm {H}(x_t,o) \le r \text { or } \mathrm {H}(x_t,o)\ge r+d \right\}$. It holds that $\Pr \left( \mathrm {H}(x_{T_{r, d}},o) \le r\right) = \mathord {\text {O}}\mathord {\left( \max \{r, \log n\}/n\right) }$ where $d = \varepsilon n$ for a not too large constant $\varepsilon > 0$.

#### Proof

We begin with considering another random sequence $y_0,y_1,y_2,y_3,\ldots$ where for each $t \in \mathbb {N}$ the point $y_t$ is created by flipping one randomly selected bit in $y_{t-1}$. Let $T^{\prime }_{r, d}$ be defined as $T_{r, d}$ but with respect to $y_t$ instead of $x_t$.

Let $p_x := \Pr \left( \mathord {\mathrm {H}}\mathord {\left( y_{T^{\prime }_{r, d}}, o\right) } \le r \mid \mathord {\mathrm {H}}\mathord {\left( y_0, o\right) } = x\right)$, i. e., the probability to enter the Hamming ball before reaching distance *d* given the process is started with Hamming distance *x*. Note that, for symmetry reasons, $p_x$ is well defined, i. e., the probability does only depend on the Hamming distance *x* and not the specific choice of $y_0$.

By definition of $T^{\prime }_{r, d}$ we have $p_x = 1$ for $x \le r$ and $p_x = 0$ for $x \ge r+d$. For all other values of *x*, i. e., for $x \in \{r+1,r+2,\ldots ,n-r-1\}$ we have

$$\begin{aligned} p_x = \left( \frac{n-x}{n}\right) p_{x+1} + \left( \frac{x}{n}\right) p_{x-1} \end{aligned}$$

by definition of the sequence $y_t$ because with probability $(n-x)/n$ the Hamming distance to the centre of the Hamming ball *o* is increased by 1 and with the remaining probability *x* / *n* it is decreased by 1. If we pessimistically assume that the probability to move towards the Hamming ball is always equal to $(d+r-1)/n$ we obtain an upper bound on $p_x$ and are in the situation of the gambler’s ruin problem with initial funds $s_a=x-r$ and $s_b=d+r-x$, $p_a = (n-d-r+1)/n$, and $p_b = (d+r-1)/n$ and the probability to be ruined

$$\begin{aligned} q(r, d, x) = \frac{\left( \frac{d+r-1}{n-d-r+1}\right) ^{x-r} - \left( \frac{d+r-1}{n-d-r+1}\right) ^{d}}{1-\left( \frac{d+r-1}{n-d-r+1}\right) ^{d}} \end{aligned}$$

gives an upper bound on the probability to enter the Hamming ball before reaching distance *d* when starting with Hamming distance *x* to the centre of the Hamming ball. We consider the probability *q*(*r*, *d*, *x*) for different values of *r*, *d* and *x*. We are interested in the results for $d = \mathord {\varTheta }\mathord {\left( n\right) }$ and consider for this $d = \varepsilon n$ where we chose the constant $\varepsilon > 0$ such that $d+r \le (n/2) - \delta n$ for some positive constant $\delta$. It is clear that due to the upper bound on *r* such a constant $\varepsilon$ exists.

It is not difficult to see that $\lim _{n\rightarrow \infty } q(r,d,r+1)=\mathord {\varTheta }\mathord {\left( (r+d)/n\right) }$. For $r=\mathord {\varTheta }\mathord {\left( n\right) }$ this is $\mathord {\varTheta }\mathord {\left( 1\right) }$ and the best bound we can obtain. For $r=\mathord {\text {o}}\mathord {\left( n\right) }$ we need to be more precise.

We begin with the case $r = \mathord {\text {o}}\mathord {\left( n\right) }$ and $r = \mathord {\varOmega }\mathord {\left( \log n\right) }$. For this setting we consider $q(r, \log n, r+1)$ and know that $\lim _{n\rightarrow \infty } q(r, \log n, r+1) = \mathord {\varTheta }\mathord {\left( r/n\right) }$ holds. Now we consider $q(r, \varepsilon n, r+\log n)$ and see that $\lim _{n \rightarrow \infty } q(r, \varepsilon n, r+\log n) = \mathord {\text {o}}\mathord {\left( r/n\right) }$ holds.

Finally, for the case $r = \mathord {\omega }\mathord {\left( \log n\right) }$, we also consider $q(r, \log n, r+1)$ and know that $\lim _{n\rightarrow \infty } q(r, \log n, r+1) = \mathord {\varTheta }\mathord {\left( (\log n)/n\right) }$ holds. Now we consider $q(r, \varepsilon n, r+\log n)$ and see that $\lim _{n \rightarrow \infty } q(r, \varepsilon n, 2r) = \mathord {\text {o}}\mathord {\left( (\log n)/n\right) }$ holds.

Together, we have $p_{r+1} = \mathord {\text {O}}\mathord {\left( \max \{r, \log n\}/n\right) }$ for all values of *r* and $d = \varepsilon n$. Since the sequence $y_t$ corresponds to ‘local mutations’ this proves the claim for random local search. We generalise the statement to the $(1+1)$ EA in the following way. We can express a standard bit mutation as a process where first a random number $k\in \{0,1,2,\ldots ,n\}$ is chosen and then *k* bits are selected uniformly at random to be flipped. The case $k=0$ does not flip any bit and can be ignored. The case $k=1$ is covered by the analysis for RLS. For larger $k=\mathord {\text {O}}\mathord {\left( \log n\right) }$ we observe that such a step is very similar to a sequence of *k* steps where exactly 1 bit is flipped. The difference does not change the limits we considered above. Since in one standard bit mutation *k* bits flip with probability $\mathord {\varTheta }\mathord {\left( 1/k!\right) }$ we can ignore steps where $\mathord {\omega }\mathord {\left( \log n\right) }$ bits flips since they contribute too little to change the asymptotic result. $\square$

#### Theorem 12

On $\mathrm {MHB}^{bn,\ell ,cn}$ with any constants $b \in (0, 1/(1+2e))$, $c>0$ and $\ell >0$ the $(1+1)$ EA with mutation rate 1 / *n* will spend only an exponentially small proportion of its time in optimal regions.

#### Proof

We assume the process starts inside the first Hamming ball, and consider the process as $(X_t)_{t\ge 0}$ as described before in Lemma 9. For the standard mutation, we have the drifts according to Eqs. 1 and 2 are $\delta = (1 - b)/2e$ and $\eta = b$. Applying Lemma 10 gives that after $r/(2\delta ) = 2ebn/(1-b) = \varTheta (n)$ time steps, whenever the center of the Hamming ball is moved, it holds that $\Pr \left( \mathrm {H}(x^{*}(t),x_\tau ) = r\right) \ge \delta /(\eta + \delta ) = (1-b)/(2(1 - b + 2eb))$. Conditioned on this event, the probability that the dynamic move $x^{*}(t+1)$ so that $x_\tau \notin \mathrm {B}_r(x^{*}(t+1))$ is $\Pr \left( l - 2Z \ge 1\right) = \Pr \left( Z \le \ell /2\right)$ where $Z \sim {{\mathrm{Hypergeo}}}(n,r,\ell )$, e.g. the number of bit positions being corrected by the dynamic (see the definition of *Z* before Lemma 9). Therefore, $\mathbf {E}\left[ Z\right] = \ell r/n = b\ell$ and by Markov’s inequality and $b < 1/(1+2e)$ it holds that

$$\begin{aligned} \Pr \left( x^{*}(t+1),x_\tau> r\right)&\ge \Pr \left( \mathrm {H}(x^{*}(t),x_\tau ) = r\right) \left( 1 - \Pr \left( Z> \ell /2\right) \right) \\&\ge \Pr \left( \mathrm {H}(x^{*}(t),x_\tau ) = r\right) \left( 1 - 2\mathbf {E}\left[ Z\right] /\ell \right) \\&\ge \frac{(1 - b)(1 - 2b)}{2(1 - b + 2eb)} =: p_1> \frac{2e - 1}{4(2e + 1)} > 0. \end{aligned}$$

When the $x_\tau$ is outside of the current Hamming ball, it follows from Lemma 11 that there is a probability of at least $p_2 = 1-\mathord {\text {O}}\mathord {\left( r/n\right) } = 1-\mathord {\text {O}}\mathord {\left( b\right) } > 0$ that the $(1+1)$ EA reaches linear Hamming distance to the Hamming ball before finding its way back to it. Application of the negative drift theorem [17] yields that the probability to find the way back into the optimal region within $2^{cn}$ steps is $\mathord {\text {O}}\mathord {\left( e^{-n}\right) }$ for a sufficiently small constant $c>0$.

We have just show that in every $\varTheta (n)$ time steps, whenever a change occurs to the target bitstring there is a probability of at least $p_1 p_2 (1 -e^{-\varOmega (n)})$ that the $(1+1)$ EA will lose track of the optimal region where $p_1$ and $p_2$ are constants. Applying this argument *n* times (a change occurs approximately every *cn* time steps), we conclude that with an overwhelmingly high probability, the $(1+1)$ EA will spend no more than $\mathord {\text {O}}\mathord {\left( n^3\right) }$ time steps within the optimal region of the $\mathrm {MHB}^{bn,\ell ,cn}$ function. $\square$

### Efficiency of Non-elitist, Population-Based Algorithms

Theorem 13, which is the main result in this section, gives conditions under which the non-elitist, population-based Algorithm 2 tracks the optimal regions of dynamic functions efficiently. We show that these conditions can be satisfied for the moving Hamming-balls function $\mathrm {MHB}^{bn,\ell ,cn}$ for any constant $b \in (0,1)$.

#### Theorem 13

If there are constants $\rho ,\delta >0$ and $\gamma _0\in (0,1)$ such that

1. *F* is a $(\lambda ,\rho )$-stable dynamic function wrt. $p_\mathrm {mut}$ with $\lambda =\varOmega (n)$, and
2. $p_\mathrm {sel}$ satisfies $\beta (\gamma )\ge \gamma (1+\delta )/\rho$ for all $\gamma \in (0,\gamma _0]$,

then Algorithm 2 initialised with $P_0\subset \mathrm {OPT}_{0}$ tracks the optima of *F* efficiently.

Condition 1 of the theorem requires that the optimal region of the function does not move too much relatively to the variation operator $p_\mathrm {mut}$ during one generation. The population size $\lambda$ is a parameter of the algorithm which can be chosen freely. So if the function is $(\kappa ,\rho )$-stable, then the first condition can be satisfied by setting population size $\lambda =\kappa$. Condition 2 requires that the selection mechanism $p_\mathrm {sel}$ induces a sufficiently high selective pressure. Note that increasingly high selective pressure is required for decreasing values of $\rho$, where $\rho$ is the probability of recovering the optimal search region via mutation (see Definition 4).

**Fig. 2:**
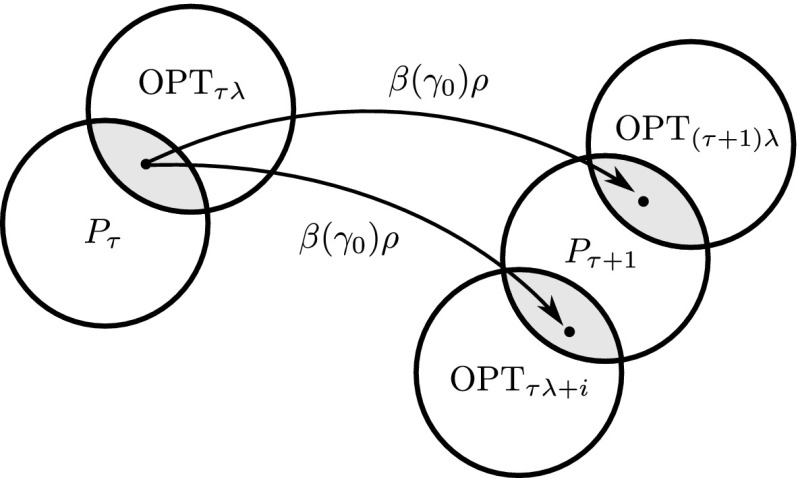
Illustration of Lemmas 14 and 15

The central argument in the analysis is illustrated in Fig. 2. It follows from the stability-assumption that any search point in $\mathrm {OPT}_{\tau \lambda }$ can be mutated into $\mathrm {OPT}_{\tau \lambda +i}$ for any $i\in [\lambda ]$ with probability at least $\rho$. Hence, if the algorithm selects a search point in $\mathrm {OPT}_{\tau \lambda }$ with probability $\beta (\gamma _0)$, then the offspring belongs to $\mathrm {OPT}_{\tau \lambda +i}$ with probability at least $\beta (\gamma _0)\rho \ge \gamma _0(1+\delta )$. This argument is invoked in both of the two steps of the analysis.

#### Lemma 14

Assume that conditions 1 and 2 of Theorem 13 hold. Then for any $\tau \in \mathbb {N}$, $i\in [\lambda ]$, if $|P_{\tau }\cap \mathrm {OPT}_{\tau \lambda }|\ge \gamma _0\lambda$, then any offspring in generation $\tau +1$ belongs to $\mathrm {OPT}_{\tau \lambda +i}$ with probability at least $\gamma _0(1+\delta )$.

#### Proof

The algorithm produces an individual in $\mathrm {OPT}_{\tau \lambda +i}$ if the algorithm selects an individual in $\mathrm {OPT}_{\tau \lambda }$ and mutates this individual into $\mathrm {OPT}_{\tau \lambda +i}$. The probability of this event is $\beta (\gamma _0)\rho \ge (1+\delta )\gamma _0$. $\square$

Lemma 15, which is the *first step* of the analysis, implies that in every generation $\tau \in \mathbb {N}$, a large fraction of the population $P_\tau$ belongs to $\mathrm {OPT}_{\tau \lambda }$. This can be shown inductively by arguing using Lemma 14 that if many individuals in $P_\tau$ belong to $\mathrm {OPT}_{\tau \lambda }$, then whp. many individuals in $P_{\tau +1}$ belong to $\mathrm {OPT}_{(\tau +1)\lambda }$. Knowing that many individuals in $P_\tau$ belong to $\mathrm {OPT}_{\tau \lambda }$ for every generation $\tau$ gives us some control on the dynamics of the population. However it does not imply that the dynamic performance measure in Definition 3 is satisfied because the individuals in $\mathrm {OPT}_{\tau \lambda }$ may not necessarily have been optimal when they were generated. A *second step* in the analysis is therefore required, showing that if sufficiently many individuals in population $P_{\tau }$ belong to $\mathrm {OPT}_{\tau \lambda }$, then many offspring in generation $\tau +1$ were optimal at the time they were generated. This second step is contained in the proof of Theorem 13.

#### Lemma 15

Assume that conditions 1 and 2 of Theorem 13 hold. Then for any generation $\tau \in \mathbb {N}$, if $|P_{\tau }\cap \mathrm {OPT}_{\tau \lambda }|\ge \gamma _0\lambda$, then

$$\begin{aligned} \Pr \left( \left| P_{\tau +1}\cap \mathrm {OPT}_{(\tau +1)\lambda }\right| \ge \gamma _0\lambda \right) \ge 1-e^{-\varOmega (\lambda )}. \end{aligned}$$

#### Proof

By Lemma 14, any offspring in generation $\tau +1$ belongs to $\mathrm {OPT}_{(\tau +1)\lambda }$ independently with probability $\gamma (1+\delta )$. Hence, by a Chernoff bound, the probability that less than $\gamma _0\lambda$ offspring belongs to $\mathrm {OPT}_{(\tau +1)\lambda }$ is $e^{-\varOmega (\lambda )}$.

We are now in position to prove the main result of this section.

#### Proof of Theorem 13

We say that generation $\tau$ *fails* if $|P_\tau \cap \mathrm {OPT}_{\tau \lambda }|\ge \gamma _0\lambda$ and $|P_{\tau +1}\cap \mathrm {OPT}_{(\tau +1)\lambda }|< \gamma _0\lambda$. By Lemma 15 and a union bound, the probability that any of the first $e^{c\lambda }/\lambda$ generations fails is $e^{-\varOmega (\lambda )}$, assuming that $c>0$ is a sufficiently small constant. By Lemma 2 and assuming no failure, any individual $x_{i}$ with $\lambda<i<e^{c\lambda }$ belongs to the optimal region $\mathrm {OPT}_{i}$ with probability at least $\gamma _0(1+\delta )$. By the definition of the algorithm, individuals within the same generation are produced independently. During any time interval $(t,t+\lambda )$ where $t,\lambda<t<e^{c\lambda }$, at least $\lambda /2$ individuals are produced in the same generation, and hence independently. It therefore holds by a Chernoff bound that for any time interval with $\lambda<t<e^{c\lambda }$,

$$\begin{aligned} \Pr \left( \sum _{i=t}^{t+\lambda }\mathbbm {1}_{\{x_i\in \mathrm {OPT}_{i}\}} \ge \gamma _0 \lambda /2\right) \ge 1-e^{-\varOmega (\lambda )}. \end{aligned}$$

The theorem now follows by taking into account the failure probability with a union bound, and choosing the parameters $t_0=\lambda , \ell =\lambda ,$ and $c^{\prime }=\gamma _0/2$ in Definition 3. $\square$

Theorem 13 implies that with a sufficiently slow dynamic, e.g. $\kappa = cn$ for any constant $c>0$, the population-based algorithm can efficiently track the moving optima of the function, given that $p_\mathrm {sel}$ induces a sufficiently strong selective pressure. We now show that given any constant $\rho \in (0, 1)$, it is possible to parameterise many selection mechanisms so that they satisfy this requirement on $p_\mathrm {sel}$. The selection mechanisms are:

- In *k-tournament selection*, *k* individuals are sampled uniformly at random with replacement from the population, and the fittest of these individuals is returned.
- In $(\mu ,\lambda )$-*selection*, parents are sampled uniformly at random among the fittest $\mu$ individuals in the population.
- A function $\alpha :\mathbb {R}\rightarrow \mathbb {R}$ is a ranking function [8] if $\alpha (x)\ge 0$ for all $x\in [0,1]$, and $\int _0^1\alpha (x)\,\mathrm {d}x = 1$. In ranking selection with ranking function $\alpha$, the probability of selecting individuals ranked $\gamma$ or better is $\int _0^\gamma \alpha (x)\,\mathrm {d}x$. *Linear ranking* selection uses $\alpha (x) := \eta (1-2x)+2x$ for some $\eta \in (1,2]$. *Exponential ranking* selection uses $\alpha (x):=\eta e^{\eta (1 - x)}/(e^\eta - 1)$ for some $\eta >0$.

The following theorem shows how these selection mechanisms can be parameterised to satisfy the second requirement of Theorem 13, and hence ensure that Algorithm 2 tracks the moving optima of any $(\lambda ,\rho )$-stable function with respect to the mutation operator $p_\mathrm {mut}$.

#### Theorem 16

For any constant $\rho \in (0,1)$, let *F* be any $(\lambda ,\rho )$-stable function wrt. $p_\mathrm {mut}$ for $\lambda =\varOmega (n)$. If there is a constant $\delta >0$ such that Algorithm 2 initialised with $P_0\subset \mathrm {OPT}_{0}$, and selection mechanism $p_\mathrm {sel}$ either

- *k*-tournament selection with $k \ge (1+\delta )/\rho$,
- $$(\mu ,\lambda )$$-selection with $$\lambda /\mu \ge (1+\delta )/\rho $$,
- linear ranking selection with $\eta \ge (1 + \delta )/\rho$, or
- exponential ranking selection with $\eta \ge (1+\delta )/\rho$,

then the algorithm tracks the optima of *F* efficiently.

#### Proof

The result follows from Theorem 13 if we can show that there exist constants $\delta ^{\prime }>0$ and $\gamma _0 \in (0,1)$ such that $\beta (\gamma )\ge (1+\delta ^{\prime })\gamma /\rho$ for all $\gamma \in (0,\gamma _0]$. The results for *k*-tournament, $(\mu ,\lambda )$-selection and linear ranking follow from Lemmas 5, 6 and 7 from [13] with $\rho$ in place of $p_0$. For exponential ranking, we notice that

$$\begin{aligned} \beta (\gamma )&\ge \int _{0}^\gamma \frac{\eta e^{\eta (1 - x)}\,\mathrm {d}x}{e^\eta - 1} = \left( \frac{e^\eta }{e^{\eta }-1}\right) \left( 1 - \frac{1}{e^{\eta \gamma }}\right) \ge 1 - \frac{1}{1 + \eta \gamma }, \end{aligned}$$

the result then follows similarly to *k*-tournament as in the proof of Lemma 5 in [13] with $\eta$ in place of *k* (Equations (3) and (4) in [13] show that $\beta (\gamma ) \ge 1 - 1/(1+\gamma k)$, then the constants $\gamma _0$ and $\delta ^{\prime }$ are shown to exist given the condition on *k*). $\square$

Finally, we apply Theorem 16 to show that population-based EAs can track the optima of the example Moving Hamming Ball function efficiently. Note that the parameter $\eta$ in linear ranking selection can only take values in the interval (1, 2]. The conditions of Theorem 16 can therefore only be satisfied if $\rho >1/2$, i.e., the optimal regions can only change slightly. For the last part of the paper, we therefore exclude linear ranking selection.

#### Corollary 17

For any constants $\delta >0, b \in (0,1)$, $c>0$, $d>0$ and $\ell \ge 1$, Algorithm 2 with the bitwise mutation operator $p_\mathrm {mut}^\mathrm {EA}$ for $\chi =1$, with population size $\lambda = cn/(2(1+d))$, and selection mechanism $p_\mathrm {sel}$ either

- *k*-tournament selection with $k \ge (1+\delta )3(\ell /b)^{\ell }$,
- $$(\mu ,\lambda )$$-selection with $$\lambda /\mu \ge (1+\delta )3(\ell /b)^{\ell }$$,
- exponential ranking selection with $\eta \ge (1+\delta )3(\ell /b)^{\ell }$.

can efficiently track the moving optima of $\mathrm {MHB}^{bn,\ell ,cn}$.

#### Proof

It follows from Lemma 7 that for any constant $\varepsilon >0$, $\mathrm {MHB}^{bn,\ell ,cn}$ is $\left( \frac{cn}{1+d}, (b/\ell )^{\ell } e^{-(1+\varepsilon )}\right)$-stable with respect to the mutation operator $p_\mathrm {mut}^\mathrm {EA}$. Since $e^{-(1+\varepsilon )}>1/3$ for a sufficiently small $\varepsilon$, the function is also $\left( \frac{cn}{1+d},(1/3)(b/\ell )^{\ell }\right)$-stable. The result then follows by applying Theorem 16. $\square$

## Conclusion

This paper has considered the frequently stated intuition that evolutionary algorithms maintaining a *population* of diverse solutions can be more resilient to dynamic changes in the objective function than algorithms maintaining single solutions. We have described a general class of fitness functions where population-based evolutionary algorithms outperform single-individual evolutionary algorithms. We have proved that for this function class, single-individual approaches, such as the $(1+1)$ EA and RLS, have a constant risk of losing the optimal solution region at any given time. Moreover, these single-individual algorithms not only lose the optimal region with constant probability, but are also likely to drift away from the optimal region subsequently.

On the other hand, assuming a not too high frequency of change, we describe sufficient conditions such that a non-elitist population-based evolutionary algorithm will remain within the optimal region with overwhelmingly high probability. Our analysis covers a range of the most commonly used selection mechanisms, and we provide appropriate parameter settings for each of them. Furthermore, the success of the population-based evolutionary algorithm does not rely on an explicit diversity mechanism. Our analysis gives further explanations of how and why populations can be essential and widely used in dynamic optimisation.

As future work, we would like to investigate further the influence of population settings within this class of dynamic functions, such as elitist populations, the necessary condition for the population size with respect to the frequency and magnitude of changes and how a population could rebuild itself after losing a few optimal solutions.

## Appendix

### Lemma 18

(Theorem 5.4 in [16]). Let $X \sim {{\mathrm{Pois}}}(\theta )$, then for all $\theta> x > 0$

$$\begin{aligned} \Pr \left( X \le x\right) \le e^{-\theta }\left( \frac{e\theta }{x}\right) ^x. \end{aligned}$$

### Lemma 19

(Inequality (3) in [21]).

$$\begin{aligned} \forall x \ge 0 \quad 1+x \le \exp \left( \frac{x}{2}\cdot \frac{x+2}{x+1}\right) . \end{aligned}$$

## References

[1] Branke, J., Wang, W.: Theoretical analysis of simple evolution strategies in quickly changing environments. In: Proceedings of the 5th Annual Conference on Genetic and Evolutionary Computation, GECCO’03, pp. 537–548. (2003)

[2] Dang, D.-C., Jansen, T., Lehre, P.K.: Populations can be essential in dynamic optimisation. In: Proceedings of the 17th Annual Conference on Genetic and Evolutionary Computation Conference, GECCO’15, pp. 1407–1414. ACM (2015)

[3] Dang D-C, Lehre PK (2016). Runtime analysis of non-elitist populations: from classical optimisation to partial information. Algorithmica.

[4] Droste, S.: Analysis of the (1+1) EA for a dynamically bitwise changing OneMax. In: Proceedings of the 2003 International Conference on Genetic and Evolutionary Computation, GECCO’03, pp. 909–921. Springer (2003)

[5] Droste S, Jansen T, Wegener I (2002). On the analysis of the (1+1) Evolutionary Algorithm. Theor. Comput. Sci..

[6] Droste S, Jansen T, Wegener I (2006). Upper and lower bounds for randomized search heuristics in black-box optimization. Theory Comput. Syst..

[7] Fu, H., Lewis, P.R., Sendhoff, B., Tang, K., Yao, X.: What are dynamic optimization problems?. In: Proceedings of the IEEE Congress on Evolutionary Computation, vol. 2014, pp. 1550–1557. (2014)

[8] Goldberg, D.E., Deb, K.: A comparative analysis of selection schemes used in genetic algorithms. In: Proceedings of the First Workshop on Foundations of Genetic Algorithms, FOGA 1991, pp. 69–93. Morgan Kaufmann (1991)

[9] Jansen, T., Schellbach, U.: Theoretical analysis of a mutation-based evolutionary algorithm for a tracking problem in the lattice. In: Proceedings of the 7th Annual Conference on Genetic and Evolutionary Computation, GECCO’05, pp. 841–848. ACM (2005)

[10] Jansen, T., Zarges, C.: Evolutionary algorithms and artificial immune systems on a bi-stable dynamic optimisation problem. In: Proceedings of the 16th Annual Conference on Genetic and Evolutionary Computation Conference, GECCO’14, pp. 975–982. ACM (2014)

[11] Kötzing, T., Lissovoi, A., Witt, C.: (1+1) EA on generalized dynamic OneMax. In: Proceedings of the 2015 ACM Conference on Foundations of Genetic Algorithms XIII, FOGA 2015, pp. 40–51. ACM (2015)

[12] Kötzing, T., Molter, H.: ACO beats EA on a dynamic pseudo-boolean function. In: Proceedings of the 12th International Conference on Parallel Problem Solving from Nature, vol. Part I, PPSN’12, pp. 113–122. Springer (2012)

[13] Lehre, P.K.: Fitness-levels for non-elitist populations. In: Proceedings of the 13th Annual Conference on Genetic and Evolutionary Computation, GECCO’11, pp. 2075–2082. ACM (2011)

[14] Lissovoi A, Witt C (2015). Runtime analysis of ant colony optimization on dynamic shortest path problems. Theor. Comput. Sci..

[15] Lissovoi A, Witt C (2016). MMAS vs. population-based EA on a family of dynamic fitness functions. Algorithmica.

[16] Mitzenmacher M, Upfal E (2005). Probability and Computing: Randomized Algorithms and Probabilistic Analysis.

[17] Oliveto PS, Witt C (2011). Simplified drift analysis for proving lower bounds in evolutionary computation. Algorithmica.

[18] Oliveto PS, Zarges C (2015). Analysis of diversity mechanisms for optimisation in dynamic environments with low frequencies of change. Theor. Comput. Sci..

[19] Rohlfshagen, P., Lehre, P.K., Yao, X.: Dynamic evolutionary optimisation: an analysis of frequency and magnitude of change. In: Proceedings of the 11th Annual Conference on Genetic and Evolutionary Computation, GECCO’09, pp. 1713–1720. ACM (2009)

[20] Stanhope, S.A., Daida, J.: (1+1) genetic algorithm fitness dynamics in a changing environment. In: Proceedings of Congress in Evolutionary Computation, IEEE CEC’99, pp. 1851–185 (1999). doi:10.1109/CEC.1999.785499

[21] Topsøe F, Cho YJ, Kim JK, Dragomir SS (2007). Some bounds for the logarithmic function. Inequality Theory and Applications.

[22] Yang, S., Yao, X. (eds.): Evolutionary Computation for Dynamic Optimization Problems, volume 490 of Studies in Computational Intelligence. Springer (2013)

